# Feasibility and limitations of bulk density assignment in MRI for head and neck IMRT treatment planning

**DOI:** 10.1120/jacmp.v15i5.4851

**Published:** 2014-09-08

**Authors:** Alexander L. Chin, Alexander Lin, Shibu Anamalayil, Boon‐Keng Kevin Teo

**Affiliations:** ^1^ Perelman School of Medicine University of Pennsylvania Philadelphia PA; ^2^ Department of Radiation Oncology University of Pennsylvania Philadelphia PA USA

**Keywords:** MRI, dose calculation, IMRT, treatment planning

## Abstract

Head and neck cancers centered at the base of skull are better visualized on MRI than on CT. The purpose of this investigation was to investigate the accuracy of bulk density assignment in head and neck intensity‐modulated radiation therapy (IMRT) treatment plan optimization. Our study investigates dose calculation differences between density‐assigned MRI and CT, and identifies potential limitations related to dental implants and MRI geometrical distortion in the framework of MRI‐only‐based treatment planning. Bulk density assignment was performed and applied onto MRI to generate three MRI image sets with increasing levels of heterogeneity for seven patients: 1) ${\text{MRI}}_{W}$: all water‐equivalent; 2) ${\text{MRI}}_{W\;+\;B}$: included bone with density of $1.53\;g/{\text{cm}}^{3}$; and 3) ${\text{MRI}}_{W\;+\;B\;+\;A}$: included bone and air. Using identical planning and optimization parameters, MRI‐based IMRT plans were generated and compared to corresponding, forward‐calculated, CT‐based plans on the basis of target coverage, isodose distributions, and dose‐volume histograms (DVHs). Phantom studies were performed to assess the magnitude and spatial dependence of MRI geometrical distortion. ${\text{MRI}}_{W}$‐based dose calculations overestimated target coverage by 16.1%. Segmentation of bone reduced differences to within 2% of the coverage area on the CT‐based plan. Further segmentation of air improved conformity near air–tissue interfaces. Dental artifacts caused substantial target coverage overestimation even on ${\text{MRI}}_{W\;+\;B\;+\;A}$. Geometrical distortion was less than 1 mm in an imaging volume $20\;\times \;20\;\times \;20\;{\text{cm}}^{3}$ around scanner isocenter, but up to 4 mm at 17 cm lateral to isocenter. Bulk density assignment in the framework of MRI‐only IMRT head and neck treatment planning is a feasible method with certain limitations. Bone and teeth account for the majority of density heterogeneity effects. While soft tissue is well visualized on MRI compared to CT, dental implants may not be visible on MRI and must be identified by other means and assigned appropriate density for accurate dose calculation. Far off‐center geometrical distortion of the body contour near the shoulder region is a potential source of dose calculation inaccuracy.

PACS numbers: 87.61.‐c, 87.55.‐D

## I. INTRODUCTION

The advent of conformal radiation therapy with intensity‐modulated radiation therapy (IMRT) has necessitated precise target delineation, allowing for improved local control by maximizing tumor dose while minimizing normal tissue exposure. In nasopharyngeal carcinoma, IMRT enhances target coverage compared to conformal 3D radiotherapy (3D‐CRT), allowing high rates of locoregional control^(1)^ without escalating the dose to normal tissues.^(2)^

Computed tomography (CT) is the standard for treatment planning because the images offer high spatial resolution and can be easily correlated with tissue electron density for accurate radiation dose calculations. However, magnetic resonance imaging (MRI) offers improved soft‐tissue contrast and target volume delineation over CT, especially for nasopharyngeal tumors, which may appear larger and more irregularly shaped on MRI than on CT.^(3)^ In addition, MRI has been found to improve evaluation of the extent of primary nasopharyngeal tumors located at the base of skull, as well as to more accurately identify soft‐tissue invasion outside of the nasopharynx. ^(4)^ Similar advantages have been described in other disease sites, such as the prostate.^(5)^

Modern treatment planning software enables CT and MRI image fusion to utilize enhanced target delineation on MRI while retaining CT electron density information. This technique is the current gold standard for head and neck treatment planning when target delineation on MRI is desired, such as for tumors located at the base of skull.^3^, ^4^ However, MRI alone has yet to supplant CT for treatment planning, given limitations such as lack of electron density information, intrinsic image distortions,^(6)^ and inability to image cortical bone and some high‐density materials. Additionally, practical limitations include longer imaging time and contraindications due to pacemakers or the presence of metals in the body.

With the advent of MRI‐guided radiation therapy,^7^, ^8^ the feasibility of using MRI directly for dose calculation is a significant area of interest. MRI‐based treatment planning was previously investigated for prostate^9^, ^10^, ^11^, ^12^ and brain targets.^(13)^ These studies utilized either atlas‐based electron density mapping^(14)^ or a segmentation technique.^(12)^ Both methods produced no significant differences in dosimetric accuracy within the pelvis compared to CT‐based planning. However, tissue heterogeneity in the head and neck can have a greater effect on dose calculation accuracy, given the presence of air and anatomical differences across individuals. Bulk density assignment on MRI for head and neck targets yields accurate dose calculation for 3D CRT planning,^(15)^ but its impact on IMRT planning has not been fully elucidated. Other efforts have focused on developing MRI sequences with ultrashort echo time that can distinguish bone from air without CT data.^16^, ^17^

Another potential limitation of MRI‐based treatment planning is intrinsic image distortion. System‐related geometrical distortion in MRI arises from the background magnetic field inhomogeneity and spatial nonlinearity of field gradients.^18^, ^19^ In radiation therapy, geometrical distortion impacts the accuracy of organs‐at‐risk (OAR) and target contours. Several studies have previously investigated the effect of MRI geometrical distortion on treatment planning using target volumes in the pelvis^20^, ^21^ or phantoms.^(22)^ One study in the pelvic region demonstrated residual image distortions of < 5 mm within 36 cm of the magnet isocenter after gradient distortion correction.^(21)^ Using 3D CRT planning on phantoms, another study demonstrated distortion‐associated variations in dose distribution of ‐8% to +4%.^(22)^ Image distortion would be expected to have less of an impact in the head and neck region than in the pelvis due to closer proximity of target volumes and organs at risk to the magnet isocenter. Nevertheless, the impact of distortion on dose distribution in the head and neck region with IMRT treatment planning remains unclear.

In addition to system related geometrical distortion, patient‐induced distortions arising from differences in magnetic susceptibility can compromise the geometrical integrity of the MRI image.^(23)^ Tissue magnetic susceptibility‐induced distortions typically manifest at air–tissue or tissue–bone interfaces. These distortions increase with MRI field strength, as well as the strength of the encoding gradient. Several studies^24^, ^25^ to quantify this distortion have been performed using numerical calculation, as well as experimental measurements, with results indicating that magnetic susceptibility distortion are smaller than system‐related distortions.^(25)^

Beyond the general limitations of MRI, a specific issue that arises in head and neck treatment planning is the presence of dental implants in many patients. On CT, these structures often have very high X‐ray attenuation coefficients, leading to streak artifacts in the areas surrounding the implant.^(26)^ With CT‐based treatment planning, these effects can be corrected for by contouring the implants and the artifacts around each implant and assigning them approximate relative electron densities. This allows more accurate modeling of the beam attenuation properties of the material. Other more advanced techniques, such as the application of a virtual filter around the teeth, have also been explored.^(27)^ In contrast, standard MRI sequences are neither able to image dental implants nor normal teeth, prohibiting the ability of MRI‐based treatment plans to model the high X‐ray attenuation of the implant material. New sequences, such as ultrashort echo time^16^, ^17^ and sweep imaging with Fourier transformation (SWIFT),^(28)^ may have utility in imaging high‐density implants, in addition to bone and teeth. However, to our knowledge, no study to date has investigated the effect of dental implants on dose calculation using MRI‐based IMRT treatment planning.

Our study sought to establish the dosimetric accuracy and to identify limitations of bulk density assignment IMRT for nasopharyngeal carcinoma in the framework of MRI‐only treatment planning. For each patient, we created three inverse‐optimized MRI‐based plans with increasing levels of segmentation: 1) all tissue assigned water‐equivalent density; 2) bulk density assignment of bone; and 3) bulk density assignment of bone and air. We then forward‐calculated each plan on CT with heterogeneity correction to measure dosimetric accuracy. To characterize some of the potential limitations of our approach, we quantified the effects of MRI geometrical distortion on dose calculation in the head and neck region. In addition, several patients had dental implants in place at the time of treatment planning, and we investigated the impact of these artifacts on dose calculation when assigning them bulk bone‐equivalent density.

## II. MATERIALS AND METHODS

### A. Data acquisition

Seven adult patients with stage III‐IV nasopharyngeal carcinoma treated with concurrent chemotherapy and IMRT were identified for this study. MRI images extended inferiorly only to the C4 vertebral body to minimize image acquisition time and to assist solely with target delineation of gross disease in the nasopharynx/base of skull. Patients were first simulated on either a PET/CT (TF Big Bore; Philips Healthcare, Andover, MA) or CT scanner (Sensation Open; Siemens Medical Solutions, Malvern, PA) with a voxel size of $1\;\times \;1\;\times \;2\;{\text{mm}}^{3}$. The same immobilization devices were used to scan the patients on the MRI (Siemens Magnetom Espree 1.5T; Siemens Medical Solutions). A six‐channel body coil was placed over the patient mask and shoulder region in place of the dedicated head coil in order to accommodate the head support and face mask. For purposes of dose calculation, we utilized a 2D axial T1 weighted spin echo sequence with a TR = 755 ms and TE = 12 ms. Vendor software 2D distortion correction option was turned on for all scans. The field of view was 26 cm with a voxel size of $0.51\;\times \;0.51\;\times \;4\;{\text{mm}}^{3}$.

### B. Contouring and segmentation

For the purpose of maintaining identical contours for use in the MRI planning and CT validation processes, CT and MRI images were rigidly registered using a mutual information algorithm in the Eclipse treatment planning software (Version 11.0; Varian Medical Systems, Palo Alto, CA). Primary site gross disease was delineated on MRI and saved onto the primary CT, while critical structures were generated directly on CT. For MRI‐based treatment planning, external body contours were generated on MRI, while internal organs‐at‐risk contours were copied from the original CT‐based treatment plan to control for potential variability in structure delineation. However, small variations in setup between the CT and MRI scans resulted in differences between the CT and MRI external contours, such that original internal contours occasionally extended beyond the MRI body. Therefore, to ensure consistency and to exclude the effects of body contour differences on the dosimetric comparison, MRI and CT external contours were matched and all internal contours were cropped 5 mm within the MRI external contour, and used in both CT and MRI images.

We accounted for differences between the extent of CT and MRI image datasets. CT data extended inferiorly to the lung apices and original treatment plans were calculated using complete CT data. To standardize MRI‐based plan comparisons, we cropped the external body contours on all CT and MRI images inferiorly at the C4 mid‐vertebral body, and internal contours were cropped 1 cm superior to this level.

Threshold segmentation with manual editing was performed on the CT to delineate bone (Hounsfield number HU > 250) and air (HU < ‐300). These new contours were saved on CT and transferred to MRI images to standardize comparisons. Four image sets were created: 1) CT with modified external and internal contours (CT); 2) MRI water only (${\text{MRI}}_{W}$); 3) MRI with bone segmentation $\left({\text{MRI}}_{W\;+\;B}\right)$; and 4) MRI with bone and air segmentation $\left({\text{MRI}}_{W\;+\;B\;+\;A}\right)$. Segmented bone was assigned bulk physical density of $1.53\;g/{\text{cm}}^{3}$ (relative electron density of 1.45), while air was assigned $0\;g/{\text{cm}}^{3}$. Remaining soft tissue on MRI was assigned water‐equivalent density.

Patients 3, 6, and 7 had dental implants in place, which required unique consideration. On CT, these implants resulted in incorrect Hounsfield numbers around adjacent tissue due to streak artifacts, and on MRI, the implants were not visualized. The exact composition of the implant material for each patient was also unknown. In the CT plans, the Hounsfield number for the implants was assigned a relative electron density of 3. This was likely an underestimate of the attenuating properties of the material, given that a common implant material is titanium which has a relative electron density of 3.8. The image artifacts around the implant were contoured and assigned water‐equivalent density. In the MRI plans, all teeth, regardless of implants, were assigned bulk bone‐equivalent density.

### C. Plan generation and comparison

For this study, a modified CT‐based, inverse‐optimized, seven‐field (nonopposing) IMRT plan (with cropped contours to account for the smaller MRI image size) was generated with the same prescribed dose and fractions, beam angles, and dose constraints as the original CT‐based plan. All plans were required to meet the maximum dose constraints and dose prescriptions detailed in Table 1. Gross disease (PTV70) received 70 Gy, intermediate‐risk regions (PTV63) 63 Gy, and low‐risk regions (PTV59) 59.5 Gy. Optimization parameters were adjusted such that all other dose constraints met in the original plan were also met in the modified CT plan.

Using the same planning and optimization parameters as the CT‐based plan, we created inverse IMRT plans on ${\text{MRI}}_{W}$, ${\text{MRI}}_{W\;+\;B}$, and ${\text{MRI}}_{W\;+\;B\;+\;A}$. To quantify the effect of heterogeneity correction on dose calculation, we used the MRI‐based inverse plans with the same monitor units and performed a forward dose calculation using the CT with heterogeneity correction. We compared dose statistics between ${\text{MRI}}_{W}$, ${\text{MRI}}_{W\;+\;B}$, ${\text{MRI}}_{W\;+\;B\;+\;A}$ and their respective CT‐based forward plans. Differences in volumes and doses were calculated by subtracting actual CT‐based values from predicted MRI‐based values.

**Table 1:** Plan constraints for key organs at risk and dose prescriptions for target volumes

*Organ At Risk*	*Dose Constraint*
Brainstem	Max. ≤ 54 Gy
Cord	Max. ≤ 45 Gy
Cord + 5 mm margin	Max. ≤ 50 Gy
Optic chiasm	Max. ≤ 54 Gy
Optic nerves	Max. ≤ 54 Gy
*Target Coverage*	
At least 95% of all PTVs should receive 100% of the prescribed dose ○ PTV70: 95% should receive 70 Gy ○ PTV63: 95% should receive 63 Gy ○ PTV59: 95% should receive 59.5 Gy At least 99% of all PTVs should receive 93% of the prescribed dose ○ PTV70: 99% should receive 65 Gy ○ PTV63: 99% should receive 58.6 Gy ○ PTV59: 99% should receive 55 Gy	

In order to assess the dosimetric impact of incorrect density assignment in the region surrounding the dental hardware in Patients 3, 6, and 7, subsets of each target volume, sPTV63 and sPTV70, were defined as the axial slices encompassing the dental implants on the CT. The DVHs of the sPTV63 and sPTV70 volumes were compared and spatial differences in dose between ${\text{MRI}}_{W\;+\;B\;+\;A}$ and their respective CT‐based forward plans were generated for analysis.

### D. Geometrical distortion

MRI geometrical distortion is an important consideration in the context of MRI‐only treatment planning. In order to ensure the spatial accuracy of contours derived from MRI, we measured the MRI geometrical distortion to assess any potential impact on dose calculation. The MRI phantom for the American College of Radiology (ACR) accreditation program was used to assess geometrical distortion. The phantom has a diameter of 20 cm, length 17 cm, and a 1.5 cm spaced square grid at the center. The phantom was scanned at isocenter and also with 10 cm longitudinal and 7 cm lateral offsets using the body and spine matrix coils. A T1 weighted 2D axial sequence (TR = 564 ms, TE = 21 ms, FOV = 25 cm, matrix = 256 × 256, 5 mm slice thickness, 17 contiguous slices) was utilized with 2D scanner distortion correction and intensity normalization turned on. A CT scan of the phantom was used as reference for the evaluation of MRI geometrical distortion. MRI and CT images of the grid were processed by first subtracting a background image without the grid, then up‐sampled to twice the resolution and smoothed with a Gaussian filter. The 109 intersection points of the grid were then extracted from the local maxima in the processed images to create landmarks for determination of geometrical distortion in the axial plane.

## III. RESULTS

### A. Impact of bulk density assignment in MRI on dose calculation accuracy

Table 2 shows the PTV coverage differences (expressed as percentage volume differences between actual CT‐based and predicted MRI‐based V100% and V93%) of each plan for various dose prescriptions. Across all seven patients, the ${\text{MRI}}_{W}$‐based plan produced the greatest PTV coverage difference, consistently over‐predicting coverage, especially of PTV70. Actual PTV70 volumes receiving 100% of the prescribed dose were 12.0%, 18.2%, 65.0%, 12.9%, 6.5%, 14.0%, and 65.5% smaller than predicted in the ${\text{MRI}}_{W}$ plan for Patients 1 through 7, respectively.

Once bone was segmented on the MRI images and assigned a bulk density value, dose calculation accuracy improved substantially. The ${\text{MRI}}_{W\;+\;B}$‐based plan resulted in less than 3% V100% and V93% over‐prediction for all patients, except for Patient 3 (Table 2) which had a substantial part of PTV70 close to the dental implants.

**Figure 1:**
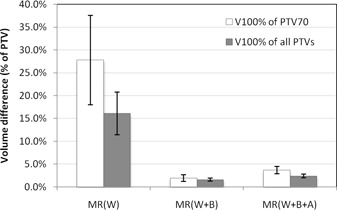
Average V100% volume differences between CT‐based and MRI‐based dose calculations. Negative differences signify smaller volumes on MRI. N = 7 for PTV70 and N = 19 for all PTVs. Error bars represent one standard error of the mean.

Segmentation of air in addition to bone did not further improve dose calculation accuracy on MRI. V100% differences for PTV70 on ${\text{MRI}}_{W\;+\;B\;+\;A}$ ranged from 1.5% to 7.4% (Table 2), slightly greater than on ${\text{MRI}}_{W\;+\;B}$. Mean V100% differences between CT‐ and MRI‐based calculations are shown in Fig. 1. Overall, actual coverage area of all PTVs (± one standard error of the mean) was 16.1% ± 4.7% smaller than predicted on ${\text{MRI}}_{W}$, while only 1.6% ± 0.3% and 2.4% ± 0.4% smaller than predicted on ${\text{MRI}}_{W\;+\;B}$ and ${\text{MRI}}_{W\;+\;B\;+\;A}$, respectively.

**Table 2:** PTV coverage differences between CT‐based and MRI‐based dose calculations. Negative differences signify smaller volumes on MRI. Note: Plans for Patients 1 and 3 did not include PTV59

	*MRI(W)*						
	*Patient 1*	*Patient 2*	*Patient 3*	*Patient 4*	*Patient 5*	*Patient 6*	*Patient 7*
PTV59 (V100%)	‐	5.3%	‐	3.5%	2.7%	3.7%	5.8%
PTV59 (V93%)	‐	0.0%	‐	0.0%	0.2%	2.2%	‐0.1%
PTV63 (V100%)	1.7%	3.6%	9.9%	8.3%	2.3%	6.5%	9.8%
PTV63 (V93%)	0.3%	0.2%	1.2%	1.7%	0.7%	1.7%	0.4%
PTV70 (V100%)	12.0%	18.2%	65.0%	12.9%	6.5%	14.0%	65.5%
PTV70 (V93%)	0.0%	0.1%	0.1%	0.0%	0.8%	0.9%	0.1%
	MRI(W + B)						
	*Patient 1*	*Patient 2*	*Patient 3*	*Patient 4*	*Patient 5*	*Patient 6*	*Patient 7*
PTV59 (V100%)	‐	1.0%	‐	‐1.8%	1.7%	1.8%	1.6%
PTV59 (V93%)	‐	0.0%	‐	0.0%	0.1%	1.6%	0.2%
PTV63 (V100%)	‐0.1%	0.7%	2.2%	1.4%	1.0%	2.4%	1.9%
PTV63 (V93%)	‐0.0%	0.1%	0.7%	1.3%	0.4%	1.0%	0.2%
PTV70 (V100%)	‐0.2%	0.9%	5.7%	0.4%	2.5%	2.2%	2.6%
PTV70 (V93%)	‐0.1%	0.0%	‐0.1%	0.0%	0.6%	0.5%	0.0%
	MRI(W + B + A)						
	*Patient 1*	*Patient 2*	*Patient 3*	*Patient 4*	*Patient 5*	*Patient 6*	*Patient 7*
PTV59 (V100%)	‐	1.6%	‐	‐0.1%	0.3%	2.6%	1.7%
PTV59 (V93%)	‐	0.0%	‐	0.0%	0.0%	1.6%	‐0.1%
PTV63 (V100%)	0.3%	‐0.3%	2.9%	2.0%	0.8%	2.0%	2.6%
PTV63 (V93%)	0.1%	‐0.4%	0.9%	0.0%	‐0.1%	1.3%	0.3%
PTV70 (V100%)	1.5%	1.7%	7.4%	3.5%	4.1%	3.7%	2.6%
PTV70 (V93%)	‐0.1%	‐0.1%	0.0%	0.0%	0.7%	0.3%	0.0%

### B. Comparison of MRI‐based and CT‐based inverse treatment plans

**Figure 2:**
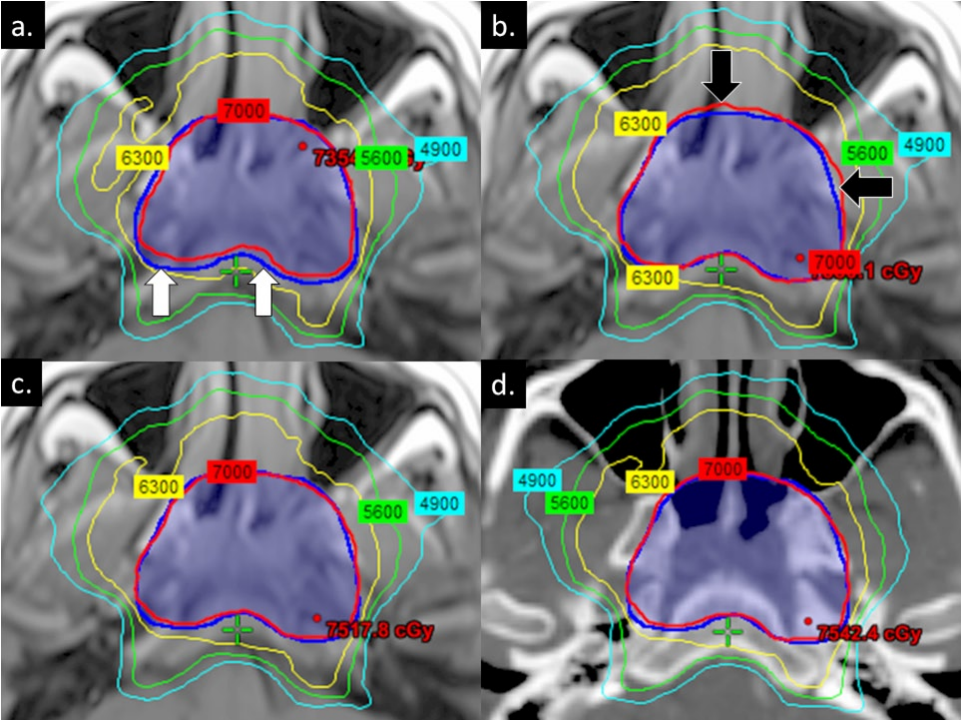
Comparison of isodose distributions between (a) ${\text{MRI}}_{W}$‐based, (b) ${\text{MRI}}_{W\;+\;B}$‐based, (c) ${\text{MRI}}_{W\;+\;B\;+\;A}$‐based, and (d) CT‐based inverse‐optimized IMRT plans for Patient 2. Images were obtained at the identical axial level based on CT‐MRI registration. Isodose distributions were calculated on CT with heterogeneity correction to standardize comparisons. Dark blue segment delineates PTV70. White and black arrows highlight areas of under‐ and overcoverage, respectively.

Figure 2 displays CT‐calculated isodose distributions of MRI‐based and CT‐based inverse‐optimized IMRT plans for Patient 2. Axial image slices at the identical level of the middle turbinate highlight the effects of bone and air on dose calculation. The inverse plan generated on ${\text{MRI}}_{W\;+\;B\;+\;A}$ most closely mirrored the CT‐based inverse plan. In contrast, the ${\text{MRI}}_{W}$‐based plan underdosed the target posteriorly, while the ${\text{MRI}}_{W\;+\;B}$‐based plan overdosed the target on the anterior and anterolateral sides.

### C. Effect of dental implants on dose calculation

**Figure 3:**
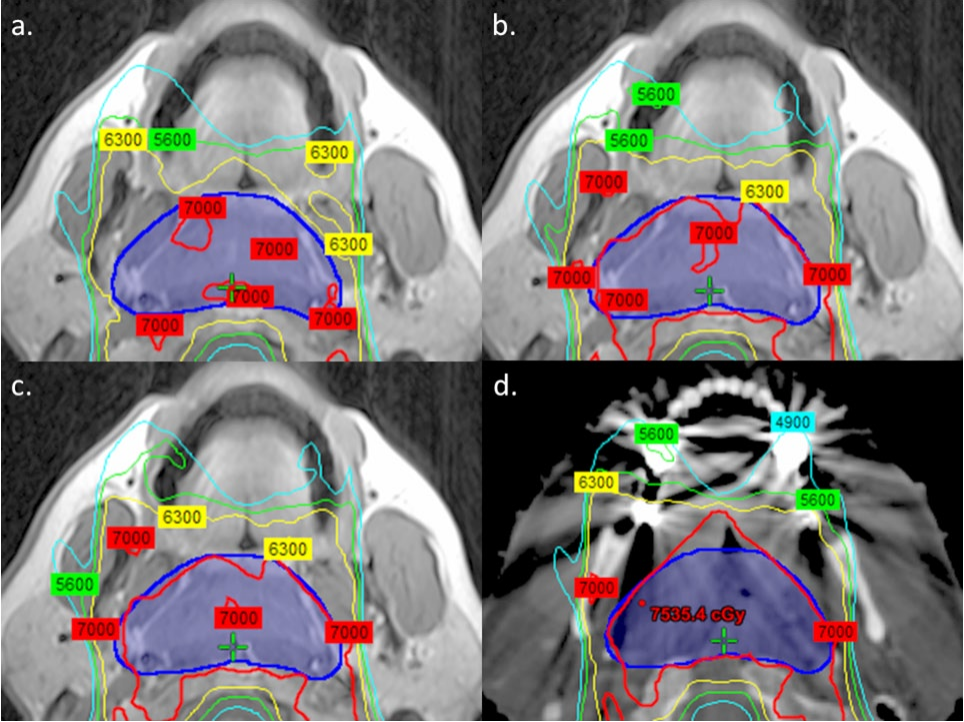
Comparison of isodose distributions between (a) ${\text{MRI}}_{W}$‐based, (b) ${\text{MRI}}_{W\;+\;B}$‐based, (c) ${\text{MRI}}_{W\;+\;B\;+\;A}$‐based, and (d) CT‐based inverse‐optimized IMRT plans for Patient 3. Images were obtained at the identical axial level based on CT‐MRI registration. Isodose distributions were calculated on CT with heterogeneity correction to standardize comparisons.

Patients 3, 6, and 7 had dental implants that affected dose calculation on MRI‐based plans. Due to the close proximity of the target to the implants, these effects were most pronounced for Patient 3. The ${\text{MRI}}_{W}$‐based plan underdosed the target by 65.0%, compared to a maximum of 18.2% for the remaining patients without any implants. Once we segmented bone, this difference was reduced to 5.7%, but still substantially higher than a maximum of 2.5% for the remaining patients. Figure 3 compares isodose distributions across plans for Patient 3, highlighting the impact of artifacts not visible on MRI. Similar effects were seen in Patients 6 and 7, but in those cases, a smaller percentage of the target volume was in the beam path that traversed the dental implant.

**Figure 4:**
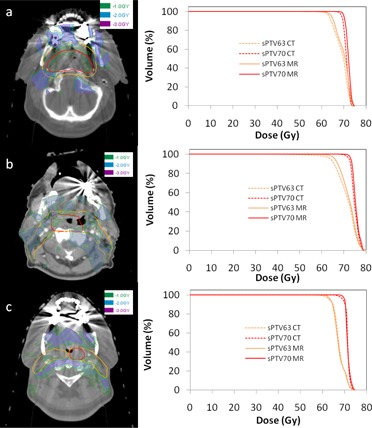
Dose difference maps between CT and ${\text{MRI}}_{W\;+\;B\;+\;A}$ plans in a 2 cm expansion volume around sPTV63 encompassing dental hardware for (a) Patient 3 (b) Patient 6, and (c) Patient 7. Red and orange contours denote sPTV70 and sPTV63, respectively. Green, blue, and purple regions indicate dose differences of above 1 Gy, 2 Gy, and 3 Gy, respectively. The corresponding DVH between the CT and ${\text{MRI}}_{W\;+\;B\;+\;A}$ plans are shown on the right.

Figure 4 illustrates the spatial regions where dose differences near the dental implants occur for Patients 3, 6, and 7. For bilateral implants, the greatest dose difference (purple contour) is located close to the implants and is located medially in the overlap region between the left and right anterior oblique IMRT fields. Comparison of the DVHs of sPTV63 and sPTV70 between the CT and ${\text{MRI}}_{W\;+\;B\;+\;A}$ plans show a lack of coverage that results in a decrease in V100% of sPTV70 and sPTV100 by 11.2% and 2.3%, respectively, for Patient 3. The corresponding values were 0.7% and 2.7% for Patient 6, and 3.7% and 1.3% for Patient 7.

### D. Geometrical distortion in the head region and the body contour

**Figure 5:**
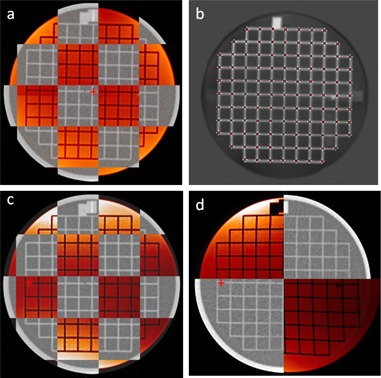
Axial CT and MRI (red) view of ACR phantom at magnet isocenter (a), processed MRI image with digitized landmarks (b), ACR phantom displaced 7 cm lateral and 10 cm longitudinal from magnet isocenter with distortion correction (c), and without distortion correction (d). Red crosshair indicates magnet isocenter axis.

**Figure 6:**
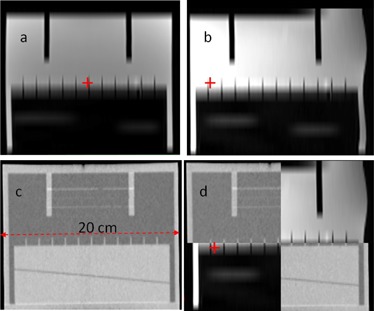
Coronal view of ACR phantom to assess geometrical distortion of body contour: (a) MRI image at magnet isocenter, (b) MRI image at 7 cm lateral displacement, (c) CT image, and (d) checkerboard fusion of MRI and CT at 7 cm lateral displacement. Red crosshair indicates magnet isocenter axis.

Figure 5(a) shows a checkerboard view of the phantom at the scanner isocenter. The result of a processed MRI image with the digitized landmarks is shown in Fig. 5(b). The mean and standard deviation of the absolute distance between corresponding MRI and CT landmarks is 0.39 ± 0.30 mm at isocenter, 0.80 ± 0.40 mm with 10 cm longitudinal offset, 0.43 ± 0.31 mm with 7 cm lateral offset, and 0.54 ± 0.34 mm with both 10 cm longitudinal and 7 cm lateral offsets. The effectiveness of vendor geometrical correction software for a phantom imaged 10 cm longitudinal and 7 cm lateral to isocenter is illustrated in Fig. 5(c) (correction on) versus (d) (correction turned off). Comparison of the coronal views of the phantom with no lateral offset in Fig. 6(a) and with 7 cm lateral offset in Fig. 6(b) indicate up to 4 mm distortion of the body contour at 17 cm lateral to isocenter.

## IV. DISCUSSION

This study demonstrates that MRI‐based treatment planning for head and neck cancers can be as dosimetrically accurate as the standard CT‐based approach. Segmenting MRI images and assigning bulk density values substantially improves accuracy. MRI‐based treatment planning without heterogeneity correction overestimated PTV coverage by 15%–30%. Accounting for bone improved target coverage on the MRI‐based plan to within 2% of the CT‐based plans. True bone and teeth densities are highly heterogeneous, but bulk value bone density assignment sufficiently accounted for most heterogeneity effects.

Further segmentation of air in addition to bone did not produce substantial improvement of target coverage over bone alone, perhaps due to the competing effects of replacing air with water. However, qualitative comparison of the isodose distributions revealed that the ${\text{MRI}}_{W\;+\;B\;+\;A}$‐based plans most closely mirrored the CT‐based plans, especially in areas with large air pockets, such as the sinonasal region. On the other hand, ${\text{MRI}}_{W\;+\;B}$‐based plans overdosed these areas, suggesting that air segmentation may improve plan conformity, even if not quantifiable in overall PTV coverage calculations.

Our results support the conclusion that bone accounts for the majority of density heterogeneity in the head and neck region. This is consistent with prior research in other target areas, such as the pelvis^(29)^ and brain.^30^, ^31^ Given complex anatomy of the head and neck, it was necessary to establish similar findings for nasopharyngeal targets.

Variation existed between patients in our study and it highlighted certain limitations in MRI‐based treatment planning. In particular, the inability of MRI to image dental implants posed a potential issue that had never been addressed in prior studies of MRI‐based planning.^9^, ^10^ We found that assigning the implant to a density equivalent to cortical bone underestimated the effect of the implant on radiation attenuation, with PTV coverage difference greater than 5% for one patient between MRI and CT. Dose differences were greatest in patients with bilateral implants and with implants located in regions where more than one anterior IMRT field traversed them.

Until new sequences are developed to directly visualize teeth and dental implants on MRI, special attention must be paid to implants in MRI‐based treatment planning. One potential solution, while laborious, is to identify implants on pretreatment dental evaluations and to manually segment them on the simulation MRI. Given the recent finding of wide dose variability to the teeth during radiotherapy, contouring individual teeth is justified and may become routine practice.^(32)^ One can also replace implants with material of known density or extract selected teeth prior to treatment. Regardless, beam angles should be selected to avoid implants, if possible. If not, beam weights should be reduced to minimize the impact of implants on dose distribution.

Intrinsic image distortion was another limitation to MRI‐based treatment planning that we addressed in the present study. Our phantom studies indicated that geometrical distortion was < 1 mm within a 20 cm diameter around the magnet isocenter axis. However, at 17 cm lateral to isocenter, we observed distortions of the body contour of up to 4 mm. We expect larger distortions further out from the scanner axis. This raises a potential problem when including the shoulder region for head and neck treatment planning. For a single 6 MV radiation field treating a depth of 6 cm, a difference of 4 mm of tissue results in 2% dose difference. The dose difference is reduced in a multifield IMRT scenario where field angles directly traversing the shoulder are generally avoided. In order to simulate such a scenario, we compared two plans where the body contour at the shoulder region differs by 4 mm. In a seven‐field IMRT configuration with two fields partially traversing the shoulder, the difference in the mean dose for the axial slices of PTV63 that includes the incorrect body contour was 0.5%.

There are several potential limitations to our study. To standardize comparisons, our technique for MRI segmentation of bone and air relied on CT data. This was necessary to validate the dosimetric accuracy of bulk density assignment on MRI using identical MRI and CT contours, the primary scope of this study. Once validated, the feasibility of MRI‐based treatment planning will rest on the ability to delineate bone, air, and soft tissue on MRI alone. Other groups have investigated the use of ultrashort echo time sequences for PET/MRI to derive an attenuation map that can distinguish cortical bone from air without needing anatomic reference data.^16^, ^17^ Research is ongoing to develop such sequences, and MRI‐based planning will likely require multiple MRI images to extract different information. The MRI images in this study had a limited field of view due to the nature of available retrospective data at our institution. The field of view can easily be expanded inferiorly, albeit with increased imaging time, but we do not anticipate that this will significantly impact MRI‐based dosimetric accuracy, due to the relative homogeneity of tissues in the neck compared to those in the sinonasal region. Lastly, our study is limited by its small sample size. While our findings were consistent across all seven patients and highlight the impact of unique characteristics like dental implants, future studies will need to analyze a larger dataset to reinforce our conclusions.

## V. CONCLUSIONS

Our study investigated the use of MRI in IMRT treatment planning for nasopharyngeal cancers. Several conclusions can be drawn from our results. MRI‐based radiation dose calculation in the head and neck region is feasible. Bone and teeth account for the majority of density heterogeneity effects relevant to dose calculation. Segmenting of air improves conformity of MRI‐based IMRT plans. Within a $20\;\times \;20\;\times \;20\;{\text{cm}}^{3}$ volume at scanner isocenter, geometrical distortion is less than 1 mm. Variation in dose calculation exists between patients, and unique factors, such as dental implants, must be taken into account and corrected for during treatment planning to minimize error. This will require multiple MR sequences to be acquired for segmentation purposes. Geometrical distortion of the body contour may be of concern for very large patients, although dosimetric effects may be small in a multifield IMRT configuration.

## Supporting information

Supplementary Material
