# Supplementary figures and images for: Feasibility and limitations of bulk density assignment in MRI for head and neck IMRT treatment planning
Source: J Appl Clin Med Phys. 2014 Sep 8;15(5):100–11. doi: 10.1120/jacmp.v15i5.4851 (PMC5711084; doi:10.1120/jacmp.v15i5.4851)

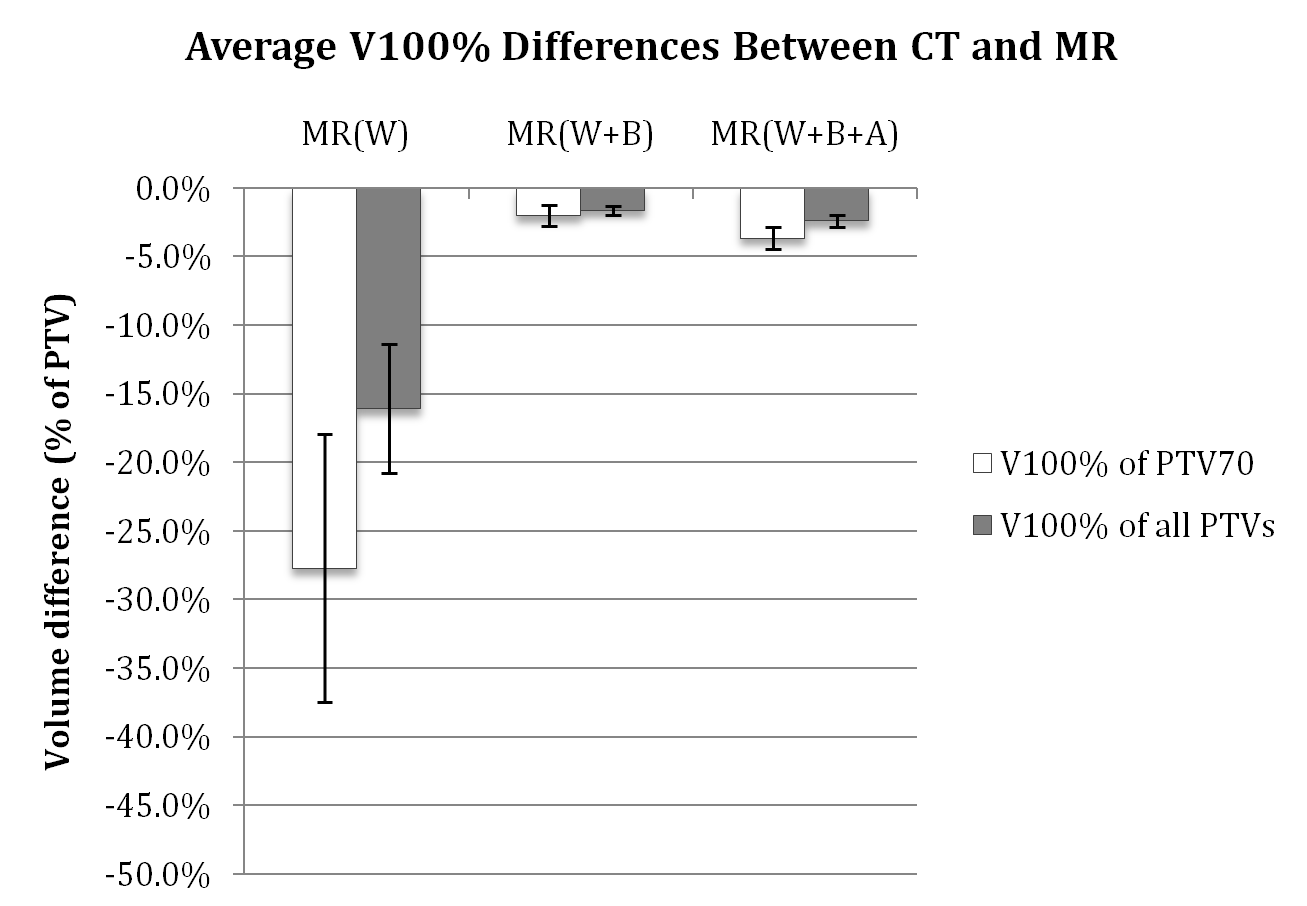

Supplement: Supplementary file 1 — Supplementary Material [file ACM2-15-100-s001.png]
